# Hypoglycemic medicines in the treatment of Alzheimer’s disease: Pathophysiological links between AD and glucose metabolism

**DOI:** 10.3389/fphar.2023.1138499

**Published:** 2023-02-23

**Authors:** Yixuan Wang, Hao Hu, Xinyu Liu, Xiangyu Guo

**Affiliations:** Dongfang Hospital of Beijing University of Chinese Medicine, Beijing, China

**Keywords:** Alzheimer’s disease, glucose metabolism, insulin signaling, mitochondria dysfunction, hypoglycemic medicines, herbal medicines

## Abstract

Alzheimer’s Disease (AD) is a global chronic disease in adults with beta-amyloid (Aβ) deposits and hyperphosphorylated tau protein as the pathologic characteristics. Although the exact etiology of AD is still not fully elucidated, aberrant metabolism including insulin signaling and mitochondria dysfunction plays an important role in the development of AD. Binding to insulin receptor substrates, insulin can transport through the blood-brain barrier (BBB), thus mediating insulin signaling pathways to regulate physiological functions. Impaired insulin signaling pathways, including PI3K/Akt/GSK3β and MAPK pathways, could cause damage to the brain in the pathogenesis of AD. Mitochondrial dysfunction and overexpression of TXNIP could also be causative links between AD and DM. Some antidiabetic medicines may have benefits in the treatment of AD. Metformin can be beneficial for cognition improvement in AD patients, although results from clinical trials were inconsistent. Exendin-4 may affect AD in animal models but there is a lack of clinical trials. Liraglutide and dulaglutide could also benefit AD patients in adequate clinical studies but not semaglutide. Dipeptidyl peptidase IV inhibitors (DPP4is) such as saxagliptin, vildagliptin, linagliptin, and sitagliptin could boost cognitive function in animal models. And SGLT2 inhibitors such as empagliflozin and dapagliflozin were also considerably protective against new-onset dementia in T2DM patients. Insulin therapy is a promising therapy but some studies indicated that it may increase the risk of AD. Herbal medicines are helpful for cognitive function and neuroprotection in the brain. For example, polyphenols, alkaloids, glycosides, and flavonoids have protective benefits in cognition function and glucose metabolism. Focusing on glucose metabolism, we summarized the pharmacological mechanism of hypoglycemic drugs and herbal medicines. New treatment approaches including antidiabetic synthesized drugs and herbal medicines would be provided to patients with AD. More clinical trials are needed to produce definite evidence for the effectiveness of hypoglycemic medications.

## 1 Introduction

Alzheimer’s disease (AD) is clinically manifested as cognitive decline and memory impairment (Reitz and Mayeux, 2014; Liu et al., 2019). It is one of the leading causes of global mortality and the most common type of dementia worldwide (Chornenkyy et al., 2019; Kumar et al., 2022b). And the prevalence of AD has been increasing steadily over the past decades (Nichols et al., 2019). The major pathological characteristics of AD are beta-amyloid (Aβ) deposits and intracellular neurofibrillary tangles (NFTs) formed by hyperphosphorylated tau protein (Grundke-Iqbal et al., 1986; Selkoe and Schenk, 2003). However, a full understanding of the mechanism is still lacking (Kumar et al., 2022a; Kumar et al., 2022b). Currently, there are still no specific drugs, despite the increasing incidence of AD and the social problems it poses (Moran et al., 2019; Srivastava et al., 2021; Michailidis et al., 2022).

Studies have demonstrated that multiple factors are associated with the onset of AD (Exalto et al., 2013; Norton et al., 2014; Andrews et al., 2021), which can be broadly classified into two categories: non-metabolic and metabolic. The non-metabolic ones include age, gender (Rahman et al., 2020), depression (Barnes and Yaffe, 2011; Feinkohl et al., 2015; Biessels and Whitmer, 2020; Srikanth et al., 2020), smoking (Barnes and Yaffe, 2011; Baumgart et al., 2015), low level of education (Exalto et al., 2013), physical inactivity (Rolandi et al., 2020), stress (Dye et al., 2017), sleep disorders (Flolingue et al., 2018), and environmental factors (Paul et al., 2018). A large amount of literature has indicated that metabolic factors such as obesity (Gendron et al., 2013; Dye et al., 2017), hyperglycemia, hypertension, and dyslipidemia (Launer, 2002; Biessels and Despa, 2018) are associated with cognitive decline.

Hyperglycemia, the main characteristic of Diabetes Mellitus (DM), is a critical metabolic risk factor in the development of AD. Researches show that DM is correlated with the development of AD (Ott et al., 1999; Butterfield et al., 2014; Natunen et al., 2020; Chae et al., 2022). T2DM and AD share common pathogenesis associated with aberrant glucose metabolism, including insulin resistance, oxidative stress, and inflammation (De Felice and Ferreira, 2014; Rosales-Corral et al., 2015; Baglietto-Vargas et al., 2016; Pugazhenthi et al., 2017; Barone et al., 2021). DM could be responsible for the progression of not only peripheral nervous system diseases but central nervous system (CNS) diseases. Abnormal metabolism in the brain is regarded as one of the main features of AD, including impaired glucose metabolism (Jack et al., 2013; Kumar et al., 2022b; González et al., 2022) and the imbalance of energy metabolism (Akhtar et al., 2016; Carvalho and Cardoso, 2021; González et al., 2022). This may eventually lead to brain lesions through a series of impaired metabolic pathways (Kellar and Craft, 2020; Michailidis et al., 2022). Dysfunction of metabolism in DM may result in neurodegeneration in the brain (Patrone et al., 2014), taking the form of Aβ accumulation and tau hyperphosphorylation as the hallmarks of AD (Grundke-Iqbal et al., 1986; Selkoe and Schenk, 2003) which is accompanied by synaptic dysfunction, neurogenesis, and neuronal disorders (Michailidis et al., 2022).

New strategies should be explored to treat AD based on its pathogenesis. Some hypoglycemic drugs that correct glucose metabolism disorders may promote brain metabolism and regeneration, and significantly improve memory and cognition (Patrone et al., 2014). Studies find that hypoglycemic drugs like Metformin, Dipeptidyl peptidase IV inhibitors (DPP4is) and glucagon-like peptide-1 (GLP-1) receptor agonists (Ras), sodium-glucose cotransporter 2 inhibitors (SGLT2is) are effective in treating Alzheimer’s disease by regulating insulin pathways and energy metabolism. Botanical medicines (Thota et al., 2020) also play an important role in regulating blood sugar and improving cognition in the treatment of both AD and DM (Ebrahimpour et al., 2020; Thota et al., 2020).

This article summarized the pathogenic relationship of AD and abnormal glucose metabolism to explain the pharmacological effects of hypoglycemic drugs and herbal medicine on AD, and aimed to provide new ideas for the treatment of AD.

## 2 Glucose metabolism and AD

Glucose homeostasis is critical to the prevention of AD. One of the tipping points for late-onset AD risk in midlife aging may be metabolic shifts (Mishra et al., 2022). The decreased glucose level in the cerebrospinal fluid (CSF) and brain can cause synaptic inactivity, which increases the chance of cognitive impairment and AD (He et al., 2022). In preclinical AD, decreased aerobic glycolysis is correlated with tau deposition (Vlassenko et al., 2018). Reduced glucose metabolism is often shown in the brains of AD patients, particularly in hippocampal and cortical regions (Wang W. et al., 2020). In post-mortem tissues of AD, the increased Blood-Brain Barrier (BBB) permeability and impaired glucose transport are observed (Sweeney et al., 2018b), and cerebral blood flow shortfall and dysfunction of the BBB may be responsible for AD (Sweeney et al., 2018a). In diabetes, high glucose levels in the plasma and other metabolic abnormalities such as hyperinsulinemia can damage the BBB and then cause hyperglycemia in CSF (Nigrovic et al., 2012; Bogush et al., 2017; Arnold et al., 2018), which can subsequently contribute to impaired insulin signaling, mitochondria dysfunction, oxidative stress, and inflammation in the brain (Szendroedi et al., 2011).

### 2.1 Insulin signaling in AD

The brain is an insulin-sensitive organ (Milstein and Ferris, 2021). Insulin plays an important physiological role in the brain except for glucose homeostasis (Duarte et al., 2012). Although the idea of synthesis of insulin in the brain is controversial (Arnold et al., 2018), it is now widely accepted that peripheral insulin can cross the BBB (Banks, 2004; Heneka and Nicotera, 2016) and bind to insulin receptors which are commonly distributed in the brain (Coll et al., 2007). Insulin signaling is involved in neuronal growth, altering synaptic plasticity (Kleinridders et al., 2014), and regulating neurotransmission (Cai W. et al., 2018), thus participating in learning, memory, and emotional functions of the brain (Roden and Shulman, 2019) by triggering the two canonical signaling cascades, the Mitogen-Activated Protein Kinase (MAPK) pathway and Akt signaling pathway (Sims-Robinson et al., 2010; Stanley et al., 2016; Arnold et al., 2018).

The abnormalities of insulin levels and insulin signaling can be significant for the onset of AD (de la Monte, 2017). In a genome-wide association study, AD was found to be correlated with insulin-related genes (Fanelli et al., 2022). Emerging evidence has demonstrated that peripheral insulin resistance is closely related to declined cerebral glucose metabolism and is a risk factor for AD in middle-aged adults (Willette et al., 2015; Femminella et al., 2021). Besides hyperinsulinemia, insulin deficiency in the plasma is involved in AD development (van der Harg et al., 2017; Imamura et al., 2020). Furthermore, disturbed insulin signaling pathways, including the Akt signaling pathway and MAPK pathway, also play an important role in the production of Aβ and phosphorylation of tau regardless of insulin levels (Goncalves et al., 2019). Starting at dysfunction of insulin receptors substrate-1 (IRS-1) (Sims-Robinson et al., 2010; Talbot et al., 2012; Gupta et al., 2018), the dysregulation of Phosphatidylinositol 3-kinase (PI3K)/Akt pathways, results in the activation of glycogen synthase kinase 3β (GSK3β) to enhance tau phosphorylation and the generation of Aβ (Phiel et al., 2003; Avila et al., 2012; Stanley et al., 2016; Dey et al., 2017; Gupta et al., 2018). The deposition of Aβ plays a role in disrupted insulin signaling, and it can compete with insulin for binding to the insulin receptor thus downregulating PI3K/Akt activation in return (Townsend et al., 2007; Kim and Feldman, 2015). Amyloid beta oligomers (AβO), a synaptic toxic formation of Aβ accumulated in the AD brain (Ferreira and Klein, 2011), can also lead to loss of insulin receptors on the cell surface, thus inhibiting the PI3K/Akt pathway (De Felice et al., 2014). What’s more, MAPK signaling participates in the phosphorylation of tau (Sims-Robinson et al., 2010), which can be proximately divided into the two subtypes, c-Jun N-Terminal Kinase (JNK) and extracellular signal-regulated kinases (ERK) (Burillo et al., 2021).

Thioredoxin-interacting protein (TXNIP) is a negative regulator of the thioredoxin (TRX) system and a modulator of oxidative stress and inflammation (Tsubaki et al., 2020). TXNIP plays an important role in AD development and may be a novel target for AD treatment. Overexpression of TXNIP in the hippocampus and cortex of AD indicated that TXNIP is related to tau and Aβ accumulation (Li et al., 2019; Ismael et al., 2021). High glucose concentration can lead to TXNIP overexpression (Nasoohi et al., 2018a). TXNIP binds to glucose transporter-1 (GLUT-1) and GLUT-4 and suppresses the glucose uptake in response to glucose evaluation. And it can mediate cerebral insulin resistance and inhibit Akt/GSK3β (Nasoohi et al., 2018b). Aβ deposition may increase TXNIP levels (Wang et al., 2019), thus promoting tau and p38 MAPK phosphorylation (Melone et al., 2018). TXNIP can also be induced by AMP-activated protein kinase (AMPK), which phosphorylates to balance energy status (Mihaylova and Shaw, 2011; Wu et al., 2013).

### 2.2 Mitochondria dysfunction

Mitochondria are essential for glucose homeostasis. Glucose metabolism is determined by mitochondria, which is regarded as a “powerhouse” (Ding et al., 2021). The tricarboxylic acid (TCA) cycle of glycolysis occurs in the mitochondria and is accompanied by energy generation. Hyperglycemia state can lead to mitochondrial calcium ion imbalance, dysregulation of the electron transport chain, and disturbed membrane potential, leading to a state of oxidative stress in mitochondria (Paul et al., 2021b). It is also revealed that blood glucose fluctuations, either hyperglycemic or hypoglycemic, can lead to oxidative stress and mitochondrial dysfunction, which is an overall concept that encompasses abnormalities in morphology and physiological function (Carvalho and Cardoso, 2021).

Mitochondria dysfunction is also a pathological link between diabetes and AD (Pugazhenthi et al., 2017). It can also interact with the pathological products of AD. Defects in mitochondrial quality control, also known as mitochondrial dysfunction could lead to cognitive dysfunction in DM (Ng et al., 2021; Luo et al., 2022). Decreased mitochondrial respiration, disrupted membrane potential, and reduced energy synthesis, which is associated with the progression of AD, cause synaptic injury in diabetic AD mice (Carvalho et al., 2015). Further studies have revealed that in transgenic AD mice, mitochondria dysfunction is related to human amyloid precursor protein (APP) which is the source of the Aβ (Ronnback et al., 2016). Aβ can also induce mitochondria dysfunction and cause metabolic disorders including impaired TCA cycle (Teo et al., 2019). An integrated metabolomics and transcriptomics study with computational models has already demonstrated that Aβ may contribute to mitochondria dysfunction and neuroinflammation by mediating oxidative stress (Butterfield and Boyd-Kimball, 2019). Moreover, tau hyperphosphorylation can trigger neurodegeneration in the diabetic retina by disrupting mitochondria function (Zhu et al., 2018).

**FIGURE 1:**
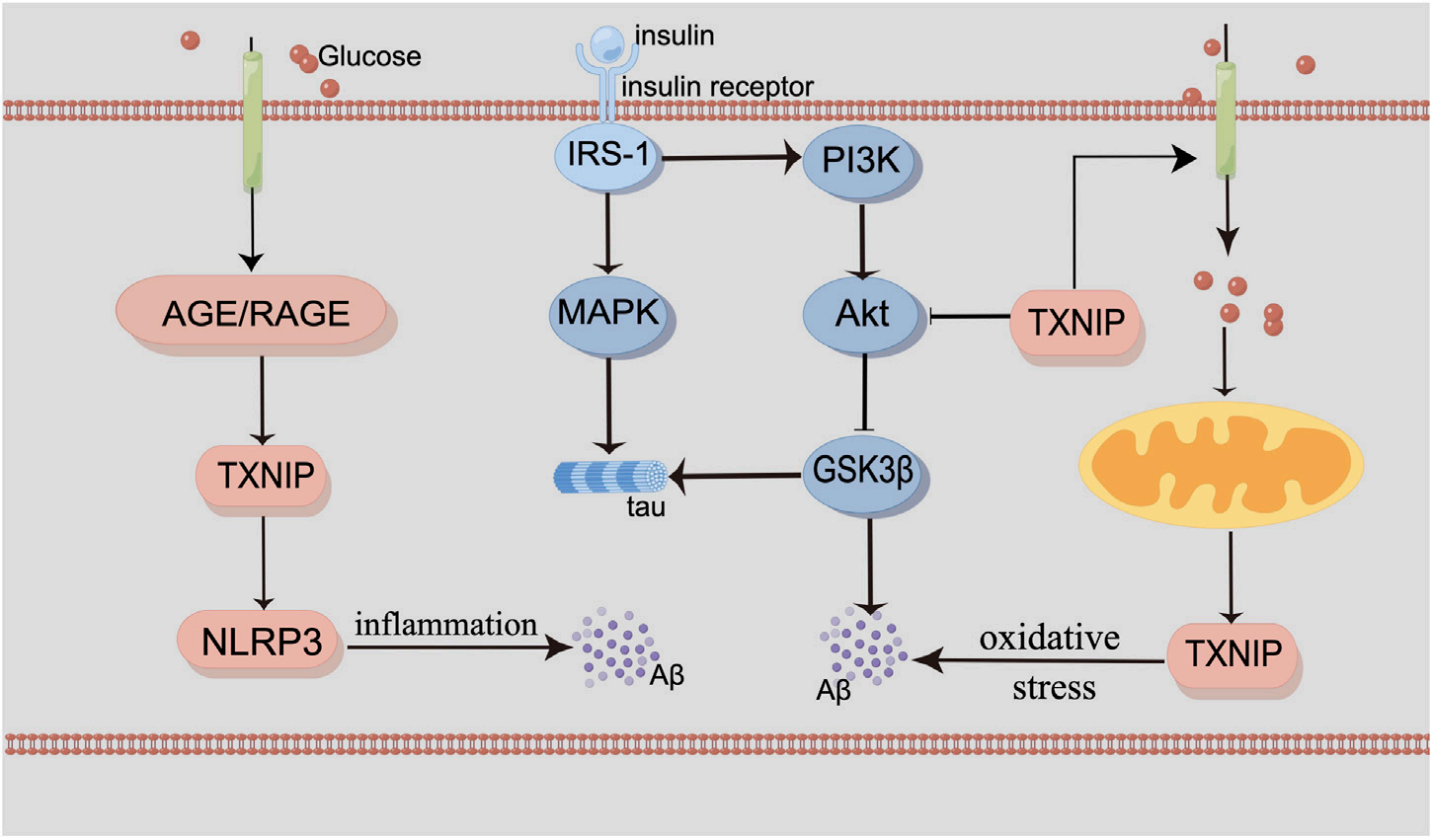
By Figdraw. The interaction of glucose metabolism and the pathogenesis of AD. Insulin binds to the insulin receptor, recruiting the IRS-1 proteins and triggering two signaling cascades, including the MAPK pathway and the PI3K/Akt/GSK3β pathway. Inhibition of PI3K and downregulation of the phosphorylated Akt protein subsequently activate GSK3β. TXNIP is a modulator of glucose metabolism and oxidative stress. It can also be induced by AMPK. RAGE/TXNIP/NLRP3 inflammasome can lead to neuroinflammation. Abnormal mitochondrial function can interact with glucose metabolism. MAPK signaling participates in the phosphorylation of tau. And the activation of GSK3β can also contribute to Aβ production and tau phosphorylation, as well as neuroinflammation and oxidative stress.

Mitochondria dysfunction is corelative with oxidative stress and inflammation, both of which are the characteristics of AD. Disruption of calcium ion homeostasis in mitochondria and mitophagy can promote the deposition of Aβ and tau (Fang et al., 2019; Jadiya et al., 2019), both of which could increase oxidative stress and energy deficiency (Kerr et al., 2017; Fertan et al., 2019). In the brain of the AD patient, oxidative stress as a result of reduced glucose metabolism could cause mitochondrial dysfunction and neuronal death (Butterfield and Halliwell, 2019). The receptor for advanced glycation end products (RAGE) is a proinflammatory ligand for advanced glycation end products (AGEs), which is also contributed to AD. RAGE/TXNIP/Nod-like receptor protein 3 (NLRP3) inflammasome is related to mitochondria dysfunction with Aβ (Figure 1).

## 3 Antidiabetic drugs

Studies have shown that some antidiabetic drugs, in addition to regulating the metabolism of peripheral tissues, are also able to modulate the metabolism of the brain, reduce inflammation and have a direct protective effect on nerves. Antidiabetic drugs may improve cognition and memory and be a viable option for AD treatment. This section of the review enumerates the possible therapeutic mechanisms for AD of metformin, GLP-1 receptor agonists, DPP4 inhibitors, insulin, and SGLT2 inhibitors, all of which are widely applied to patients with diabetes according to official Clinical Guidelines.

### 3.1 Metformin

Metformin, the most important medicine for treating people with type 2 diabetes, can lower blood glucose levels by inhibiting hepatic gluconeogenesis and increasing glucose absorption in peripheral tissues (Patrone et al., 2014). It quickly penetrates the BBB (Łabuzek et al., 2010; Ying et al., 2014), and is disseminated to several brain regions (Łabuzek et al., 2010). Metformin enhanced hippocampal-dependent and non-dependent cognitive function in prediabetic rats by lowering brain inflammation, microglia hyperactivity, brain mitochondrial dysfunction, necrosis, and tau hyperphosphorylation (Jinawong et al., 2020). Metformin also decreases brain mitochondrial dysfunction, apoptosis, Aβ aggregation, and inflammation during ischemia in non-diabetic rats following cardiac ischemia/reperfusion (Benjanuwattra et al., 2020). Metformin, aspirin, simvastatin, and angiotensin-converting enzyme inhibitors (ACEI) were called antidiabetic polypill (PP). They could ameliorate learning and memory deficits in AD or AD-T2DM mice. PP treatment improved metabolic parameters, ameliorated brain atrophy and neuronal loss, rescued synaptic loss, reduced Aβ levels, and tau phosphorylation (Infante-Garcia et al., 2018). Metformin improved cognitive deficits in the hippocampus of the APP/PS1 mice model of AD *via* promoting Chaperone-mediated autophagy (CMA)-activated degradation of Aβ, which suggested that it might be a possible therapy for AD (Xu et al., 2021).

Metformin not only regulates metabolism in peripheral tissues but also prevents the onset and development of AD by alleviating metabolic abnormalities. In high glucose-incubated HT22 cells and brains of db/db mice with cognitive dysfunction, metformin stimulated AMPK to enhance autophagic activity. It reduced hyperphosphorylated tau and improved cognitive dysfunction (Chen et al., 2019). By triggering the mammalian target of rapamycin (mTOR) signaling in elderly ApoE3-target replacement (TR) mice, metformin increased the expression of postsynaptic proteins, thus enhancing cognition (Zhang et al., 2019). Intranasal metformin produced higher metformin concentrations in the hippocampus and lowered plasma concentrations, which enhanced insulin sensitivity more than oral treatment in mice with intracerebroventricular-streptozotocin (ICV-STZ)-induced AD (Kazkayasi et al., 2022). In SH-SY5Y cells and Transgenic mice (Tg6799), metformin exacerbated the pathogenesis of AD by promoting β-APP processing in autophagosomes to boost the production of Aβ (Son et al., 2016). In transgenic *Caenorhabditis elegans* with low levels of constitutive pan-neuronal expression of human Aβ1-42 (GRU102), metformin reversed Aβ-induced metabolic defects, normalized mitochondrial function, reduced protein aggregation, and repaired defects that affect lifespan. Changes in the TCA cycle metabolism were identified even at low levels of Aβ expression by combining metabolomics, transcriptomics, and computational modeling (Teo et al., 2019).

Clinical studies have also revealed the relationship between metformin and cognitive decline. In a retrospective investigation, metformin was connected to better memory function in older people who were cognitively healthy, which suggested that metformin was connected to a lower risk of dementia (Wu et al., 2020). Metformin improved cognitive performance and decreased the risk of dementia in individuals with T2D compared to those who did not use oral antidiabetic medications, according to a population-based investigation of seniors (65 years or older) (Cheng et al., 2014). A paired study revealed that metformin might reduce hippocampus and cortical atrophy to cognitive improvement. Higher levels of Aβ and lower levels of tau and phosphorylated tau (p-Tau) in CSF were observed in patients with metformin (Pomilio et al., 2022). Results from a randomized controlled trial suggested that metformin improved cognitive function in patients with non-dementia vascular cognitive impairment (NDVCI) and abnormal glucose metabolism. Possible mechanisms were the improvement in IR and attenuation of intima-media thickness (IMT) progression (Lin et al., 2018). A prospective open-cohort study showed that metformin use was associated with a slower decline in Mini-Mental State Examination (MMSE) scores over time. The findings suggest that metformin might have a direct neuroprotective effect on participants (Secnik et al., 2021).

However, some studies suggested metformin, as a side effect, was found to possibly expedite the development of AD. A population-based nested case-control study revealed a link between the use of metformin and a higher risk of AD in newly diagnosed diabetic patients (Ha et al., 2021). A prospective research showed that metformin might be a factor in cognitive impairment, while calcium and vitamin B12 supplements helped relieve the metformin-induced vitamin B12 deficit and improved cognitive results (Moore et al., 2013). According to a study on experimental animals, metformin elevated APP and Aβ in mice, impairing the mitochondrial function of brain neurons. Additionally, metformin might directly interact with Aβ, causing Aβ aggregates to accumulate, primarily in the cortex region. Apoptotic neurons were seen in the cortical area where metformin-induced Aβ aggregates were concentrated (Picone et al., 2016). Furthermore, in APP/PS1 mice with tau seeding, metformin was found to ameliorate autophagic damage in microglia and promote phagocytosis of NP tau. This suggested that metformin could reduce Aβ load and NP tau aggregation by enhancing autophagic activity in microglia (Chen et al., 2021). But in aged APOE TR mice, metformin also enhanced tau phosphorylation, suggesting that there might be adverse implications in humans (Zhang et al., 2019). Metformin activated signaling pathways that at least partially included AMPK to influence the transcription of β-secretase (BACE1), which then catalyzed APP cleavage and significantly increased the production of Aβ (Chen et al., 2009). In LAN5 neuroblastoma cells, metformin increased APP and presenilin levels, which raised APP cleavage and intracellular accumulation of Aβ (Picone et al., 2015). It remains controversial whether metformin is a promising agent for AD. Further research should be undertaken to elucidate.

### 3.2 GLP-1 receptor agonists

The hypoglycemic effects of the hormone GLP-1 are increasing insulin secretion, decreasing glucagon secretion, delaying stomach emptying, increasing satiety, and increasing insulin sensitivity in a range of tissues (Amer Diabet, 2013). GLP-1 Ras play a crucial role in maintaining glucose homeostasis in the body (Nuamnaichati et al., 2020). Currently, some animal studies provide evidence that GLP-1 Ras improve cognitive impairment, but clinical evidence is still lacking. We have reviewed the pharmacological effects of several GLP-1 Ras agonists on AD, including short-acting GLP-1 Ras, long-acting GLP-1 Ras, a GLP-1/Glucose-dependent insulinotropic polypeptide (GIP) Dual-Agonist, and a triple GLP-1/GIP/glucagon receptor agonist (TA).

#### 3.2.1 Exendin-4/exenatide

Exendin-4 and exenatide are short-acting GLP-1 Ras. Exenatide is a synthetic product of exendin-4 (Nielsen et al., 2004). In a rat model of AD, exenatide might inhibit the inflammation response and increase cholinergic activity to protect hippocampus neurons and improve cognitive impairment (Solmaz et al., 2015). Exendin-4 can cross the BBB (Holscher, 2014; Salameh et al., 2020) and protect neurons against apoptosis induced by AβO in differentiated human neural progenitor cells (Velmurugan et al., 2012). In presenilin-1 knock-in (PS1-KI) mice, exenatide might improve cognition by increasing anaerobic glycolysis in the brain (Bomba et al., 2013). Exendin-4 treatment might prevent AD-related tau hyperphosphorylation by regulating the insulin signaling pathway and enhance memory and cognition in the brain of rats (Xu et al., 2015). Exendin-4 intervention increased PI3K/Akt activity and decreased GSK3β activity in rats (Chen et al., 2012). In the brains of type 2 diabetic rats, exendin-4 also increased protein kinase A (PKA), increased cyclic guanosine monophosphate (cGMP), increased AMPK levels, and decreased JNK activation. Furthermore, exendin-4 protected neural cells against apoptosis in the rat brain by regulating several autophagic markers (Candeias et al., 2018). In transgenic (Tg) mice, exendin-4 prevented the impaired insulin signaling in the hippocampus and enhanced cognition, as demonstrated by a decline in hippocampal serine phosphorylation of IRS-1 (IRS-1pSer) and activation of JNK. It could inhibit AβO-induced neuronal pathologies observed *in vitro* (Bomfim et al., 2012). Exenatide enhanced the long-term memory ability of adult wild-type mice, stimulated the Brain-Derived Neurotrophic Factor (BDNF)-tropomyosin-related kinase B receptor (TrkB) neurotrophic axis, and prevented apoptosis by reducing p75-neurotrophin-receptor-mediated signaling (Bomba et al., 2018). Exenatide treatment altered cognitive deficits in a triple transgenic mice model of AD (3xTg-AD) undergoing a high-fat diet by improving BDNF signaling and reducing inflammation (Bomba et al., 2019). However, the outcomes of a pilot study showed no trends in improvement in cognition, but a reduction of Aβ42 in extracellular vesicles (EV) was observed (Mullins et al., 2019).

#### 3.2.2 Liraglutide

Liraglutide, a short-acting GLP-1 Ras, can cross BBB (Hunter and Hoelscher, 2012; Hoelscher, 2014). Recent experiments have proven the direct neuroprotective effect of liraglutide on cognitive function and neuron growth. Liraglutide exerted a neuroprotective role by triggering cytoprotective growth factors on the human neuroblastoma cell line SH-SY5Y during methylglyoxal stress (Sharma et al., 2014). The application of liraglutide enhanced late-phase long-term potentiation (L-LTP), synaptic plasticity (LTP) and spatial memory in rats (McClean et al., 2010; Han et al., 2013). Liraglutide also reduced overall Aβ plaques and inflammatory responses and increased the number of young neurons in the dentate gyrus (McClean et al., 2011). In addition, liraglutide has neuroprotective properties through the regulation of pathways related to glucose metabolism. It was demonstrated that liraglutide protected against diabetes-induced neuronal loss and synaptic ultrastructure degradation in the hippocampus of DM mice with cognitive impairment and might promote autophagy *via* the AMPK/mTOR pathway in primary cultured mice hippocampal neurons (Kong et al., 2018). Peripherally injected liraglutide prevented tau phosphorylation by promoting Akt activity and decreased GSK3β activity in db/db mice (Ma et al., 2015). Liraglutide greatly decreased insulin receptor aberrations, amyloid plaque load, and glial cell activation in APP(SWE)/PS1d9E mice (Long-Smith et al., 2013). Intravenous liraglutide improved AD-like learning and memory deficits, lowered the level of tau protein hyperphosphorylation, and reduced neurodegeneration, by strengthening JNK and ERK signaling pathways and boosting o-glycosylation of neuronal cytoskeletal proteins in STZ mice (Xiong et al., 2013). Intraperitoneally administered liraglutide relieved brain chronic inflammatory responses in mice by lowering the number of activated microglia and activated astrocytes (Parthsarathy and Hoelscher, 2013). Besides, female 3xTg-AD mice given chronic liraglutide treatment, reduced the cortical Aβ1-42, partially decreased inflammatory and oxidative stress in both plasma and brain, and partially normalized brain estradiol, GLP-1 content, and PKA levels (Duarte et al., 2020).

A randomized study also demonstrated that liraglutide restored blood-brain glucose transfer capacity at BBB (Gejl et al., 2017). In another randomized, placebo-controlled double-blinded clinical trial, liraglutide blocked the decline of glucose metabolism in the brain of AD, although no significant differences were presented in Aβ load and cognition measures to the placebo with PET of [18F]fluoro-2-deoxyglucose ([18F]FDG) metabolism (Gejl et al., 2016). In 12 weeks of a placebo-controlled study, liraglutide significantly improved the results of functional magnetic resonance imaging (fMRI). Intrinsic connectivity within the default mode network (DMN) was improved in the liraglutide group relative to placebo (Watson et al., 2019).

#### 3.2.3 Other GLP-1 receptor agonists

Both lixisenatide and Val(8) GLP-1 could permeate the BBB (Gengler et al., 2012; Hunter and Hoelscher, 2012; Hoelscher, 2014), and protect LTP (Gengler et al., 2012; McClean and Hoelscher, 2014). They helped reduce the amount of amyloid plaque and tau hyperphosphorylation in AD mice (Li et al., 2012; Cai H.-Y. et al., 2018). The short-acting GLP-1 Ras lixisenatide significantly decreased neuroinflammation (McClean and Hoelscher, 2014) *via* activating PKA-CREB signaling and inhibiting p38-MAPK pathway. In a randomized, double-blind placebo-controlled trial, dulaglutide treatment in long term may improve cognition in patients with type 2 diabetes (Cukierman-Yaffe et al., 2020). Semaglutide, a long-acting GLP-1 receptor agonist, had a protective role against Aβ in AD by enhancing autophagy and inhibiting apoptosis (Chang et al., 2020).

GLP-1/GIP Dual-Agonist-5 (DA5-CH) can enhance memory function in AD mice by metabolism pathways. In the hippocampus of APP/PS1 mice, DA5-CH decreased hippocampal amyloid senile plaques and phosphorylated tau protein, increased levels of p-PI3K and p-Akt growth factor kinase, and prevented excessive activation of p-GSK3β (Cao et al., 2018). TA therapy could significantly correct the memory deficits in APP/PS1 mice, which elevated BDNF levels and further boosted neurogenesis in the dentate gyrus. TA decreased the overall level of Aβ as well as oxidative stress and neuroinflammation in the cortex and hippocampus (Kim et al., 2018).

### 3.3 DPP4 inhibitors

Diabetes treatment with DPP4 is linked to higher GLP-1 levels (Lambeir et al., 2008). Many studies have proven that DPP4is could benefit cognitive function such as saxagliptin, vildagliptin, linagliptin, and sitagliptin.

Saxagliptin and vildagliptin not only boosted GLP-1 levels in the hippocampus but corrected cognitive impairments, as well as decreased levels of Aβ42 and p-tau in the animal model. Moreover, TNF-α and IL-1β, two important cytokines produced by activated microglia, were decreased to reduce inflammation after the treatment of the two DPP4is (Kosaraju et al., 2013a; Kosaraju et al., 2013b).

Linagliptin and sitagliptin also alleviated cognitive impairments and amyloid load in AD model besides raising GLP-1 levels in the brain. Linagliptin raised GIP incretin levels and decreased tau phosphorylation caused by the downregulation of GSK3β in the brain of AD mice. It also ameliorated neuroinflammation by significantly reducing glial fibrillary acidic protein (GFAP) (Kosaraju et al., 2017). In APP/PS1 animals, sitagliptin therapy protected synaptic plasticity in AD mice by markedly activating GLP-1 and BDNF-TrkB signaling (Dong et al., 2019). It might restore damaged insulin signaling in cultured human neuronal cells, thus preventing tau hyperphosphorylation and GSK3β activation. The formation of intracellular reactive oxygen species (ROS) and mitochondria dysfunction brought on by Aβ were mitigated by sitagliptin, which may be related to the activation of 5′AMPK-Sirt1 signaling (Kornelius et al., 2015). But a conflicting view held that Sitagliptin was ineffective on tau phosphorylation in OLETF type 2 diabetic rats and instead exacerbated it, possibly through the activation of GSK3β. Additionally, sitagliptin raised ser-616 phosphorylation of IRS-1, which suggested elevated insulin resistance in the brain (Kim et al., 2012).

Clinical studies supported the findings of some experimental studies. A cross-sectional study revealed that enhanced dipeptidyl peptidase IV (DPP4) activity and lower BDNF activity were linked to a higher risk of moderate cognitive impairment (MCI) in older persons. However, it remained uncertain whether DPP4 could reduce BDNF ratio **(**DBR) by targeting increased plasma DPP4 activity, thereby improving MCI in older patients with type 2 diabetes (Zheng et al., 2018). In a prospective and observational study, older diabetic individuals who took sitagliptin for 6 months showed enhanced cognitive performance (Isik et al., 2017). A retrospective investigation revealed that DPP4i usage was linked to a low amyloid load and improved long-term cognitive outcomes in diabetic patients with Alzheimer’s disease-related cognitive impairment (Jeong et al., 2021). DPP4is showed a protective effect against the risk of dementia in Alzheimer’s disease, according to a meta-analysis (Zhou et al., 2020). In individuals with diabetes and dementia, DPP4is improved global cognition as measured by the MMSE, according to a prospective open-cohort study (Secnik et al., 2021). In a retrospective investigation, DPP4i usage was linked to slower rates of memory loss in AD patients. Additionally, DPP4is were more advantageous for APOE4 carriers (Wu et al., 2020).

### 3.4 SGLT2 inhibitors

SGLT2 inhibitors prevent the kidneys from reabsorbing glucose, which lowers blood glucose levels (Vallon and Thomson, 2017). Empagliflozin, a selective SGLT2 inhibitor, significantly prevented cognitive decline in db/db mice. This protection of cognitive abilities was linked to a reduction in brain oxidative stress and an increase in brain-derived neurotrophic factors (Lin et al., 2014). Dapagliflozin inhibited ROS-dependent neuronal apoptosis, increased the expression of PI3K/Akt/GSK-3β pathway, and suppressed neuroinflammation by reducing the activation of nuclear factor-kappa B (NF-κB) pathway and TNF-α levels to exert neuroprotective effects (Arab et al., 2021).

A retrospective study showed that when compared to DPP4is, the use of SGLT2 inhibitors was considerably more protective in T2DM patients against new-onset dementia, but not against new-onset AD (Mui et al., 2021). T2DM individuals who used SGLT2 inhibitors had a lower chance of developing dementia according to a retrospective study. The lowest risk of dementia was shown in dapagliflozin, followed by empagliflozin, while canagliflozin had no association (Wu et al., 2022).

### 3.5 Insulin therapy

The effects of insulin on cognitive impairment are debatable. Aβ-induced loss of spatial memory was prevented by intrahippocampal insulin injections (Ghasemi et al., 2014). A single insulin injection reversed additional brain Aβ accumulation and cognitive deficits in a mice model of AD induced by a high-fat diet (Vandal et al., 2014). By shifting APP processing to non-amyloidogenic pathways, intranasal insulin improved cognitive impairments and decreased brain Aβ levels and Aβ plaque deposition in APP/PS1 mice (Mao et al., 2016). Intranasal insulin improved verbal memory and acutely increased plasma Aβ42 levels. However, further research has to be done to determine the clinical implications of changed plasma Aβ42 levels (Reger et al., 2008). Intravenous insulin infusion decreased Aβ42 clearance but increased Aβ42 accumulation in plasma, which has been demonstrated in the past to cause peripheral insulin resistance (Swaminathan et al., 2018). A meta-analysis also revealed that patients with T2DM under treatment with insulin presented the lowest risk of dementia compared with other antidiabetic drugs (Zhou et al., 2020). (Table 1)

**TABLE 1:** Overview of animal studies of diabetic drugs in treatment of AD.

Diabetic drugs	Object of study	Brief cohort description	Outcome characteristics	References
Metformin	Male rats	HFD-fed rats were assigned into three subgroups with vehicle, nec-1, or metformin for 8 weeks.	Cognitive function, synaptic plasticity, dendritic spine density, microglial morphology, brain mitochondrial function, brain insulin sensitivity↑; hyperphosphorylated-tau and necroptosis↓	Jinawong et al.
Metformin	Wistar rats	Metformin 200 mg/kg was given intravenously to the cardiac I/R group, either during ischemia (D-MET) or at the onset of reperfusion (R-MET).	glucose level, IR,GSK3β, and JNK↓, GLP-1, insulin-like growth factor-1, AMPK, and PI3K/Akt↑	Benjanuwattra et al.
Metformin	APP/PS1xdb/db mice	AD-T2DM mice were received polypills in the diet from 4 to 26 weeks of age.	Learning and memory deficits, brain atrophy and neuronal loss; metabolic parameters↑; synaptic loss, Aβ, and p-tau↓	Infante-Garcia et al.
Metformin	APP/PS1 mice	APP/PS1 mice were treated with tap water with Metformin freely for 12 weeks.	cognition, CMA-activated degradation of Aβ↑	Xu et al.
Metformin	db/db mice, High glucose-cultured HT22 cells	Twelve-week old male db/db mice received consecutive intraperitoneal injection of 200 mg/kg/d metformin or (and) 10 mg/kg/d chloroquine for 8 weeks.	Cognition, autophagy activity in an AMPK dependent manner↑; hyperphosphorylated tau↓	Chen et al.
Metformin	Human ApoE (huApoE)-TR homozygous mice of the C57BL/6J background	Thirteen-month-old female ApoE3-TR and ApoE4-TR mice were respectively randomized into metformin group and normal saline (NS) group	mTOR signaling and tau phosphorylation↑	Zhang et al.
Metformin	C57BL/6 mice	ICV-STZ-injected mice were treated with intranasal or oral metformin for 4 weeks.	Learning and memory functions, phosphorylated insulin receptor and pAkt↑	Kazkayasi et al.
Metformin	transgenic *Caenorhabditis elegans* strain	Treated with metformin	normalized lifespan ↑; Aβ-induced metabolic defects, and protein aggregation↓	Teo et al.
Metformin	SH-SY5Y cells Tg6799 AD model mice	Fourteen week-old female Tg6799 were injected daily with 200 μL intraperitoneal metformin or isotonic sodium chloride for 9 days.	gamma-secretase activity, Aβ generation, autophagy and AMPK↑; mTOR↓	Son et al.
Metformin	APP/PS1 mice	Treated with metformin	autophagic activity in microglia↑; Aβ load and NP tau aggregation↓	Chen et al.
Metformin	C57BL/6 mice	Treated with metformin	APP and Aβ↑; mitochondrial function of brain neurons↓	Picone et al.
Metformin	N2a695 cells	Pretreated with metformin for 24 h	Aβ, AMPK and BACE1 ↑	Chen et al.
Metformin	Sprague Dawley albino male mature rats	Following stereotaxic surgery, STZ received rats were treated with either saline or exenatide 20 mu gr/kg/day through intraperitoneally for 2 weeks.	Cholinergic activity and cognition↑; the inflammation response↓	Solmaz et al.
Exendin-4	Human neuroprogenitor cells	Pre-incubation of neurons with exendin-4	Exendin-4 protected the neurons from apoptosis induced by Ab oligomers and stimulated cyclic AMP response element binding protein phosphorylation	Velmurugan et al.
Exenatide	PS1-KI mice 3xTg-AD mice	Daily injection of exenatide (500 mg/kg BW) or saline for 5 days a week.	In PS1-KI mice: long-term memory performances, brain lactate dehydrogenase activity, and lactate levels ↑; no effects were observed on mitochondrial respiration. In 3xTg-AD mice: exenatide had no effects on brain metabolism	Bomba et al.
Exendin-4	Goto-Kakizaki (GK) rats	Exendin-4 was continuously administered for 28 days, *via* s.c. Implanted micro-osmotic pumps (5 mu g/kg/day; infusion rate 2.5 mu L/h).	GLP-1, insulin-like growth factor-1 (IGF-1), AMPK, and PI3K/Akt↑; glucose level, IR,GSK3β, and JNK↓	Candeias et al.
Exendin-4	Sprague-Dawley rats	T2DM rats were injected with exendin-4 for 28 consecutive days.	PI3K/Akt↑, GSK3β, and tau hyperphosphorylation↓	Xu et al.
Exendin-4	PC12 cells Wistar rats	Administered exendin-4 (10 μg/kg body weight;) by a twice-daily subcutaneous injection for 14 days.	GSK3β and tau hyperphosphorylation↓	Chen et al.
Exendin-4	APP/PS1 mice	Intraperitoneal injections of exendin (25 nmol/kg, dissolved in saline) for 3 weeks.	IRS-1 and JNK↑	Bomfim et al.
Exenatide	Adult wild-type mice	2-month treatment with exenatide	Long-term memory performances, and the BDNF-TrkB neurotrophic axis↑; apoptosis↓	Bomba et al.
Exenatide	3xTg-AD mice	administered exenatide (500 μg/kg body weight) *via* intraperitoneal injection 5 days per week.	BDNF signaling↑; inflammation↓	Bomba et al.
Liraglutide	SH-SY5Y cells	Cells were pre-treated with 10, 100, and 200 nM Liraglutide	Cell viability and Akt↑; cytotoxicity and apoptosis↓	Sharma et al.
Liraglutide	Sprague–Dawley rats	Dissolved peptide (0.05–5 nmol liraglutide and/or 4 nmol Aβ25–35) solution (4 μL) was injected into the bilateral hippocampus.Control rats received only saline.	The Aβ(25–35)-induced impairment of spatial memory, deficit of L-LTP↓	Han et al.
Liraglutide	APP/PS1 mice	Injected liraglutide at 25 nmol/kg body weight intraperitonially for 8 weeks.	Young neurons in the dentate gyrus and LTP↑; memory impairments, synapse loss deterioration of synaptic plasticity, Aβ, inflammation↓	McClean et al.
Liraglutide	C57BL/6 mice	Treated with liraglutide (200 μg/kg) or vehicle for 8 weeks.	Autophagy and AMPK/mTOR↑; cognitive impairment, neuronal injuries, and ultrastructural damage to synapses↓	Kong et al.
Liraglutide	APPswe/PS1dE9 mice with a C57BL/6 background	Liraglutide treatment for 8 weeks at 25 nmol/kg body weight i.p. Once daily	IRS-1↑; IR aberrations, Aβ, astrocytosis, and microglial↓	Long-Smith et al.
Liraglutide	Kunming mice	Injected with liraglutide (300 μg/kg) for 30 days. The control and AD model groups were injected with sterile 0.9% saline.	Learning and memory ability, tau bound to microtubules↑; hyperphosphorylation levels of tau and neurofilament proteins↓	Xiong et al.
Liraglutide	C57BL/6 mice	Treated with either 0.9% NaCl or 25 nmol/kg liraglutide intraperitonially for 30 days.	The activated microglia load, the activated astrocyte load, the pro-inflammatory cytokine levels of IL-6, IL-12p70, IL-1 β, and total nitrite concentration↓	Parthsarathy and Hoelscher
Liraglutide	10-month-old 3xTg-AD female mice	Treated for 28 days with liraglutide (0.2 mg/kg)	Cortical Aβ(1–42), brain estradiol and GLP-1, oxidative/nitrosative stress and inflammation↓	Duarte et al.
Lixisenatide	APP/PS1/tau female mice	Injected lixisenatide for 60 days at 10 nmol/kg i.p.	PKA-CREB signaling pathway↑; Aβ, NFTs, neuroinflammation and p38-MAPK↓	Cai et al.
(Val(8))GLP-1	Wistar rats	Treated with STZ (i.c.v.), or treated with STZ+(Val8)GLP-1 i.c.v.	Learning and memory↑; tau, hyperphosphorylated tau, damaged cell nuclei and nucleolus↓	Li et al.
Semaglutide	SH-SY5Y cell line	Treated by semaglutide.	Autophagy↑; apoptosis↓	Chang et al.
DA5-CH	APP/PS1 mice	Injected DA5-CH intraperitoneally at a dose of 10 nmol/kg for 28 days.	Working-memory and long-term spatial memory, L-LTP, and activation of the PI3K/AKT↑; Aβ and p-tau↓,	Cao et al.
TA	APP/PS1 transgenic mice	Mice were injected with TA (10 nmol/kg body weight) i.p. Or saline for 2 months.	The anti-apoptotic signaling molecule Bcl-2, BDNF, synaptophysin, and neurogenesis in the dentate gyrus↑; memory deficit, the mitochondrial pro-apoptotic signaling molecule BAX, Aβ, neuroinflammation, and oxidative stress↓	Kim et al.
Saxagliptin	Wistar rats	Orally administered with Saxagliptin (0.25, 0.5 and 1 mg/kg) for 60 days.	GLP-1↑; Aβ, tau phosphorylation and inflammatory markers↓	Kosaraju et al.
Vildagliptin	Wistar rats	Orally administered with vildagliptin (2.5, 5 and 10 mg/kg) for 30 days.	Memory retention↑, GLP-1 levels↑; Aβ↓, tau phosphorylation↓, inflammatory markers↓	Kosaraju et al.
Linagliptin	3xTg-AD mice	Administered linagliptin orally (5, 10, and 20 mg/kg) for 8 weeks.	Cognitive deficits present↑, brain incretin levels↑; Aβ↓, tau phosphorylation↓, neuroinflammation↓	Kosaraju et al.
Sitagliptin	APP/PS1 mice	The APP/PS1 mice received daily gastric gavage administration of sitagliptin (20 mg/kg) for 8 weeks.	Cognition function, LTP, GLP-1, BDNF-TrkB signalings↑	Dong et al.
Linagliptin	SK-N-MC human neuronal cells	Exposed to 10–100 μM of linagliptin for 24 h	Aβ-induced cytotoxicity↓, GSK3β and tau hyper-phosphorylation↓, Aβ-induced mitochondrial dysfunction and intracellular ROS generation↓	Kornelius et al.
Sitagliptin	OLETF T2DM rats	Given standard rat food or rat food supplemented with 100 mg/kg/day sitagliptin for 12 weeks.	Pathological tau phosphorylation GSK3β and ser-616 phosphorylation of IRS-1↑	Kim et al.
Empagliflozin	db/db mice	Treated the standard diet containing 0.03% empagliflozin for 7 days, 10 weeks, and 16 days respectively	Cardiovascular injury, vascular dysfunction, and cognitive decline↓	Lin et al.
Dapagliflozin	Wistar rats	Rotenone (1.5 mg/kg) was subcutaneously administered every other day for 3 weeks. Dapagliflozin (1(mg/kg)/day, by gavage for 3 weeks)	PI3K/AKT/GSK-3βpathway and glial cell line-derived neurotrophic factor↑; ROS-dependent neuronal apoptosis, neuroinflammation, NF-κB pathway, and TNF-α↓	Arab et al.
Insulin	Adult male Sprague-Dawely rats	Insulin treatment (0.5 or 6 mU) for 6 days.	Aβ-induced memory deterioration, hippocampal caspase-3, ERK and P38 activation↓	Ghasemi et al.
Insulin	3xTg-AD mice	An intravenous injection of insulin (3.8 units/kg of human insulin) or saline.	Memory ability↑; soluble Aβ levels↓	Vandal et al.
Insulin	APPswe/PS1dE9 mice	6 weeks of Intranasal insulin treatment in APPswe/PS1dE9 mice.	Cognitive deficits, Aβ production and plaque formation↓	Mao et al.
Insulin	Wild-type mice The immortalized human cerebral microvascular endothelial cell line	Saline (100 µLl) or insulin (1IU) was administered *via* internal carotid artery. Cells were pre-treatmented with 100 mIU/mL insulin on the luminal side for 20 min.	Upon peripheral insulin administration in wild-type mice: The plasma clearance of Aβ40↑, the plasma-to-brain influx of Aβ40↑, the clearance of intracerebrally injected Aβ42↑; the plasma clearance of Aβ42↓, the plasma-to-brain influx of Aβ42 ↓, the clearance of intracerebrally injected Aβ40↓ In hCMEC/D3 monolayers exposed to insulin: the luminal uptake and luminal-to-abluminal permeability of Aβ40↑, the abluminal-to-luminal permeability of Aβ42 permeability↑; the abluminal-to-luminal permeability of Aβ40↓, the luminal uptake and luminal-to-abluminal permeability of Aβ42↓ Aβ cellular trafficking machinery was altered.	Swaminathan et al.
Liraglutide exendin (9–39)amide	Wistar rats	5 µL of peptides solution were injected icv.	LTP↑; exendin (9–39)amide: LTP↓	McClean et al.
Lixisenatide, Liraglutide	APP/PS1 mice	Ten weeks of daily i.p. Injections with liraglutide (2.5 or 25 nmol/kg) or lixisenatide (1 or 10 nmol/kg) or saline	Performance in an object recognition task↑; LTP, Aβ, chronic inflammation response↓	McClean and Hoelscher
Liraglutide, Insulin	db/db mice	Daily injected subcutaneously with liraglutide, insulin, or saline.	Liraglutide injection: CSF insulin↑; hyperphosphorylation of tau protein↓ insulin injection had no effects on CSF insulin or phosphorylation of tau protein.	Ma et al.
Metformin, Insulin	LAN5 neuroblastoma cells	Treated with 12.5, 25, 50, 100 and 200 mM of metformin (SIGMA) in serum free medium for 24 and 48 h, and with 0.1, 1 and 2.5 mM of metformin for 5 or 10 days.	Metformin: APP, presenilin, Aβ, oxidative stress, and mitochondrial damage ↑ Insulin: Aβ, oxidative stress, mitochondrial dysfunction, and cell death↓	Picone et al.

Symbol explanation: “↑” means upregulating, increasing, improving and so on. “↓” means downregulating, decreasing, impairing and so on.

## 4 Herbal medicines

Plants can be used as dietary supplements or as drug composition for the prevention and treatment of diseases. Phytochemicals possess abundant biological properties, and the active components of some plants are also very useful in the treatment of diseases. Numerous studies have found that phytoconstituents including plant extracts and active components have antioxidant, anti-inflammatory, and hypoglycemic properties, which can target abnormal enzyme activities and thus have a therapeutic effect on diseases. This section of the review enumerates the therapeutic value of plants, their extracts, and active metabolites for both AD and DM. Considering the link between abnormal glucose metabolism and AD, phytomedicines can be used as a promising drug treatment for AD by modulating glucose metabolism.

### 4.1 Curcumin

Curcumin is a polyphenol derived from the rhizomes of Curcuma longa (Thiruvengadam et al., 2021). It is an antioxidant and has anti-inflammatory effect. (Bradford, 2013). It has been reported that curcumin may be adopted as a potential neuroprotective agent against cognitive decline or AD (Caruso et al., 2022). Moreover, curcumin can also reverse aberrant glucose metabolism to alleviate memory decline. In AD mice, curcumin can upregulate PI3K/Akt pathways while decreasing the expression of insulin receptors and insulin receptor substrates in the hippocampus (Feng et al., 2016; Wang et al., 2017). Daily dietary supplementation with curcumin may also improve abnormal glucose metabolism and reduce the risk of diabetes and AD. Curcumin can also decrease the activation of the TXNIP/NLRP3 inflammasome to prevent cell death (Zhang M. et al., 2021). Using the micro-positron emission tomography (PET) technique in AD mice, it has been revealed that curcumin can improve glucose metabolism (Wang et al., 2017). In a high-fat diet rat model, curcumin nanoparticle (CurNP) can improve Aβ accumulation and tau hyperphosphorylation in the hippocampus through alleviating redox and inflammation and downregulating MAPK/ERK pathway (Abdulmalek et al., 2021). In a 12-week randomized controlled trial, participants with prediabetes who consumed 180 mg of curcumin daily had significantly reduced GSK3β and IAPP in serum samples after 12 weeks compared with placebo groups, reducing insulin resistance and the risk of developing AD and DM (Thota et al., 2020). In a randomized, double-blind, placebo-controlled trial, healthy older people who took solid lipid curcumin had significantly better memory performance and mood (Cox et al., 2015). A randomized, placebo-controlled, double-blind, pilot clinical trial in 6 months has shown that curcumin can help patients with AD decrease the accumulation of Aβ in the plasma (Baum et al., 2008).

### 4.2 Resveratrol

Resveratrol is a polyphenolic phytocompound, derived from various plants, including fruits (e.g., blueberries, blackberries), and herbal medicine (e.g., Polygonum cuspidatum herb) (Gambini et al., 2015). It has benefits in both lifespan and health span (Bhullar and Hubbard, 2015). Previous studies have shown that resveratrol can act as an anti-diabetic drug to decrease plasma glucose levels, and increase insulin sensitivity (Berman et al., 2017). Resveratrol is used in treating age-related diseases such as AD because of its anti-inflammatory and antioxidant propertities (Li et al., 2018). Moreover, resveratrol also can permeate the BBB and be used as a potential neuroprotective agent (Wicinski et al., 2020). A clinical study of older adults has shown that a supplement of resveratrol could improve memory function and increase hippocampal functional connectivity and glucose metabolism (Albani et al., 2010; Witte et al., 2014). It has been demonstrated that resveratrol can regulate glucose metabolism and inhibit tau phosphorylation through the PI3K/Akt pathway, respectively (Ghafouri-Fard et al., 2022). The mechanism of resveratrol in the treatment of AD and DM is the same in the SIRT1 and AMPK pathways (Meng et al., 2020). But there is insufficient evidence that resveratrol can treat AD merely by modulating glucose metabolism (Ribeiro et al., 2022). Resveratrol also had the ability to inhibit the inflammatory factors and the TXNIP/TRX/NLRP3 signaling pathway, both of which were simulated by Aβ (Feng and Zhang, 2019; Zhang M. et al., 2021). Resveratrol consumption could protect mitochondrial function, which reduces the risk of developing AD and DM. Carrasco-Pozo etc. al have found that resveratrol could affect mitochondria dysfunction and oxidative stress induced by indomethacin *in vitro*, although quercetin but not resveratrol is the most effective one (Carrasco-Pozo et al., 2012). In addition, resveratrol can promote autophagy and treat AD and DM *in vivo* and *in vitro* (Shakeri et al., 2020).

The treatment with resveratrol, however, has its drawbacks. A recent study has shown that Resveratrol could upregulate autophagy in the prefrontal cortex, but BDNF and synaptophysin decreased after the treatment of resveratrol in mice (Yang et al., 2019). Although dietary supplementation with resveratrol reduced the risk of developing AD, the bioavailability of resveratrol and the level of resveratrol in the brain were reduced in diabetic rats. This may be related to diabetic-induced osmotic diuresis (Chen T.-Y. et al., 2017). A randomized, double-blind, placebo-controlled trial has shown that the treatment of resveratrol had no benefits in biomarkers of AD and glucose metabolism. And brain volume loss was increased in the resveratrol group. This further prevents resveratrol from being a potential therapeutic agent to regulate both peripheral and central glucose metabolism.

New technological applications for resveratrol can provide broader prospects for drug formulations of resveratrol. Because of its limited bioavailability, micronized resveratrol formulations can effectively solve this problem (Singh et al., 2019). Besides, both resveratrol and its analog pterostilbene have the common stilbene backbone, as well as pleiotropic actions. Therefore, stilbene-based drug development is also an emerging direction for the treatment of AD (Giacomini et al., 2016).

### 4.3 Berberine (BBR)

BBR is an isoquinoline alkaloid extracted mainly from Berberis aristata (shrub). It has a broad spectrum of treatments such as anti-inflammatory, antitumor, lipid-lowering properties, etc (Yao et al., 2015). BBR can be used as an anti-diabetic agent by regulating peripheral glucose metabolism (Lan et al., 2015; Yao et al., 2015). Since the BBB is favorably permeable to BBR, it can also be used in the treatment of central nervous system disorders (Behl et al., 2022). In recent years, it has also been shown that BBR can improve central glucose metabolism and insulin signaling to ameliorate AD-related pathological lesions. *In vitro*, BBR induced increased glucose utilization in neurons and inhibited the production of oligomer Aβ by activating the PI3K/PGC epsilon pathway, thereby reducing AD-related neuronal damage (Wu et al., 2021). BBR can modulate mitochondria dysfunction to regulate energy metabolism in the AD cell model. It also reduces pro-inflammatory cytokines in activated microglial cells (Wong et al., 2021). In a high-fat diet diabetic rat model, BBR enhanced cognitive function by improving both peripheral and central IR, and reduced hippocampal phosphorylation of tau as well as neuronal apoptosis (Zhang J.-H. et al., 2021). BBR also inhibited insulin resistance in the medial prefrontal cortex and downregulated PI3K/Akt/mTOR and MAPK signaling pathways in diabetic rats (Chen Q. et al., 2017). In addition, in a combined rat model of AD and T2DM, BBR played a neuroprotective role by suppressing endoplasmic reticulum (ER) stress, and also attenuated memory deficits (Xuan et al., 2020). Nevertheless, due to the limited pharmacokinetic characteristics, the combination of nanotechnology and BBR is still a promising therapeutic approach (Behl et al., 2022).

Some other alkaloids can also modulate glucose metabolism for the treatment of AD. Peganum harmala (P. harmala) is an Egyptian herbal medicine, whose extract can also decrease hippocampal Aβ, phosphorylated tau, and GSK-3β (Saleh et al., 2021). Another study (Li et al., 2017) has shown that the alkaloids of P. Harmala, including harmine and harmaline (Saleh et al., 2021), were the most active components. Huperzine A (Hup A) is a Lycopodium alkaloid extracted from the moss Huperzia serrata. A recent study has shown that Hup A treatment can improve metabolism and cognition by upregulating the insulin and phosphorylated Akt levels in the cortex of high fat-induced mice, although the same results were not seen in ob/ob mice (Wang H.-y. et al., 2020).

### 4.4 Glycosides

Glycosides, especially saponins, are an important group of phytochemicals due to their diverse pharmacological activities. The therapeutic effects of these active components have received much attention in recent years. Geniposide is extracted from the fruit of Gardenia jasminoides Ellis and has various pharmacological properties (Ma et al., 2019). Primary studies have suggested that geniposide had a promising ability to treat AD by reducing amyloid plaques, inhibiting tau phosphorylation, and preventing memory impairment (Liu et al., 2015). It can also be used in managing the pathological alterations of AD by targeting glucose metabolism-related pathways such as GLP-1 receptors and insulin signaling. Geniposide can stimulate the GLP-1 receptor, which not only has an effect on diabetes but promotes neurogenesis and cognitive function *via* correcting metabolism (Sun et al., 2021). Two studies have revealed that geniposide enhanced the effect of insulin signaling on AD-related changes (Zhang et al., 2015; Zhang et al., 2016). In the diabetic AD mouse model, geniposide significantly decreased the phosphorylated level of tau directly. What’s more, the phosphorylation of GSK-3β was decelerated *in vivo*, and the phosphorylation of Akt was increased *in vitro* cells (Zhang et al., 2016). Geniposide also upregulated the levels of BACE1 and insulin-degrading enzyme (IDE), both *in vivo* and *in vitro* (Zhang et al., 2015).

Some favorable results of sapogenins have been shown in recent studies. Sarsasapogenin is extracted from the Anemarrhena asphodeloides Bunge (rhizome). It could inhibit Aβ overproduction and tau hyperphosphorylation by upregulating BACE1 and inactivating of Akt/GSK-3β cascade respectively *in vivo* and *in vitro* (Zhang Y.-M. et al., 2021). Asiaticoside, found in *Centella asiatica* (L.) Urban, also could remarkedly improve the cognitive deficits in diabetic rats by modulating oxidative stress, PI3K/Akt/NF-kappa B pathway, and synaptic function (Yin et al., 2015). Ginsenoside is an active compound of Panax ginseng C. A. Meyer or Panax quinquefolium L. (American Ginseng). In the STZ-injected mice model, ginsenoside Rb1 can enhance insulin sensitivity. At the same time, this study also revealed that it improved memory and cognition (Yang et al., 2020), which could have benefits in diabetic encephalopathy, as well as AD. Ginsenoside Rg2, steroid glycoside extracted from Panax ginseng could induce autophagy in the AD mice model, thus improving cognitive behaviors and metabolism (Fan et al., 2017).

### 4.5 Quercetin

Quercetin is a natural flavonoid from a variety of plants, including fruits and vegetable (Miltonprabu, 2019). Moreover, quercetin has beneficial effects on many diseases including diabetes and AD (Costa et al., 2016). Quercetin may protect cells from mitochondria dysfunction and oxidative stress *in vitro*, which was the most efficient compared with rutin, resveratrol, and epigallocatechin gallate (Carrasco-Pozo et al., 2012). This may reveal a joint role of quercetin in the treatment of abnormal glucose metabolism and AD-related cognitive impairment by targeting mitochondria. Subsequent studies found that quercetin improved cognitive function and aberrant peripheral glucose metabolism in db/db mice. What’s more, quercetin increased the expression of brain-derived BDNF and SIRT1 protein, which indicated a neuroprotective role of quercetin (Hu et al., 2020). A recent study aimed to identify the potential target of quercetin suggested that quercetin may have a synergic effect on DM and AD by targeting MAPK pathways (Zu et al., 2021).

Although quercetin has pleiotropic effects and a few negligible adverse effects, its low bioavailability may be a roadblock preventing it from being a therapeutic agent. Nanoparticles with quercetin may solve this problem, which has been already applied in cancer treatment (Ebrahimpour et al., 2020). Future studies could focus on improving the bioavailability of quercetin through nanotechnology and other pharmaceutical technologies.

Other flavonoid components also have great therapeutic value. Emerging studies have shown that luteolin and its derivatives (Choi et al., 2014b; Liu et al., 2014), scutellarin (Chledzik et al., 2018), and apigenin as well as its derivatives (Choi et al., 2014a), had properties of anti-diabetes and anti-AD. Luteolin treatment in high-fat diet mice, significantly improved the central insulin resistance and peripheral glucose metabolism, to alleviate cognitive decline for AD and diabetes induced by obesity (Liu et al., 2014). Some flavonoids can also reduce the overexpression of TXNIP and activation of NLRP3 inflammasome (**Zhang M. et al., 2021** **).** Notably, regarding the structure-activity relationships of flavonoids, isomers of flavonoids also play an influential role in the treatment of AD and DM, such as isomeric C-glycosylated derivatives of luteolin and apigenin (Choi et al., 2014a; Choi et al., 2014b). This may indicate that future phytochemical investigations could focus on the structure-activity relationships to clarify the pharmacological effects.

### 4.6 Herbal plants and extracts

Many natural products have inhibitive effects on both AD and diabetes. Herbal medicine has been used for thousands of years since ancient times in many countries such as China, India, and some Africa countries. Modern pharmaceutical research has also provided evidence for the application of these botanicals in the treatment of diabetes and AD. Non-etheless, plenty of research indicated that the effects of herbal plants or their extracts on diabetes and AD were associated with the inhibition of enzymes instead of modulating pathological processes. For example, many plants and extracts showed a high capacity to inhibit acetylcholinesterases (AChE) and butyrylcholinesterase (BuChE), alpha-glucosidase, and alpha-amylase (Ali et al., 2015; Zengin et al., 2015; Trampetti et al., 2019; Mahomoodally et al., 2020; Floris et al., 2021). However, most herbal plants and their extracts may belong to the restorative category, so the treatment may not delay the progress of AD (Brimson et al., 2020). But several studies have revealed the therapeutic role of herbal plants in rodent models of diabetic cognitive decline or diabetes-related AD. On the one hand, herbal plants and exacts can improve cognitive impairment in diabetic rats. Piperine can improve the cognition of diabetes rats and reduce the expression of AD-associated genes (Kumar et al., 2021). Artemisinin reduced the formation of Aβ plaques in the hippocampus of rats with central and peripheral STZ injections (Poorgholam et al., 2021). Mangifera indica Linn extracts reduced central inflammation and atrophy in the hippocampus and cortex. The metabolic parameters (body weight, glucose, and insulin levels) were reduced after the treatment of Mangifera indica Linn extracts in the diabetes mice model (Infante-Garcia et al., 2017). Withania somnifera (L.) Dunal (Solanaceae) can enhance peripheral glucose metabolisms such as insulin sensitivity and glucose uptake. It can also have properties against Aβ induced toxicity and APP (Paul et al., 2021a). Rhinacanthus nasutus (L.) Kurz (Acanthaceae) or its compound could be beneficial for diabetes and neurodegenerative diseases like AD (Brimson et al., 2020). Propolis and its extracts can ameliorate diabetic symptoms and improve glucose metabolism in the animal model. Propolis also prevented neurons from oxidative stress (Zulhendri et al., 2021).

Recent studies have further identified the therapeutic effects of some herbal plants on AD through modulating central glucose metabolism. Lychee seed extract decreased the level of Aβ and tau formation, as well as insulin resistance and AGE in the hippocampus of diabetes rats (Tang et al., 2018). Yuzu (Citrus junos Tanaka) extract also had a neuroprotective role in the hippocampus of AD rats and also attenuated hippocampal insulin signaling (Yang et al., 2013). (Table 2 and Table 3).

**TABLE 2:** Overview of animal studies of herbal medicines in treatment of AD.

Herbal medicine	Object of study	Cohort description	Sample type	Outcome characteristics	References
Curcumin	APPswe/PS1dE9 double transgenic mice	Administration of curcumin for 6 months to the HDC, MDC and LDC with diferent dose-level as 400 mg/kg/day, 200 mg/kg/day, 100 mg/kg/day	Hippocampus tissues	PI3K, p-PI3K, AKT, p-AKT↑; IR, IRS-1↓	Feng et al.
Curcumin	APPswe/PS1dE9 double transgenic mice	treated with different dose-level as 100 mg/kg/day, 200 mg/kg/day, 400 mg/kg/day. In the positive control group, 10 mg/kg/day RSG was used for treatment.	Hippocampus tissues	Cerebral glucose uptake, IGF-1R, IRS-2, PI3K, p-PI3K, Akt, p-Akt↑; IR, IRS-1↓	Wang et al.
Curcumin	Male Wistar rats	orally with curcumin, zinc sulfate, two doses of CurNP and ZnONP, as well as metformin, for 6 weeks.	Hippocampus tissues and blood sample	Aβ, p-tau, redox, inflammation, MAPK/ERK pathway↓	Abdulmalek et al.
Quercetin	The human intestinal epithelial cell line Caco-2	Caco-2 cells were incubated in INDO for 20 min	Mitochondria	Mitochondria dysfunction, oxidative stress↓	Carrasco-Pozo et al.
Resveratrol	Male C57BL/6J mice	HFD mice were treated with/without MET (250 mg/kg/day), RESV (100 mg/kg/day), or COMBO (MET: 250 mg/kg/day, RESV: 100 mg/kg/day) for 5 weeks.	The center and right prefrontal cortex and hippocampus samples	Autophagy↑; BDNF, synaptophysin↓	Yang et al.
Resveratrol	The Zucker diabetic fatty (ZDF) rat model	ZDF rats and their LN were dosed with a SGP Mixture consisting of grape seed extract, concord grape juice and RES by oral gavage for 10 days	Plasma, urine and brain tissues	The bioavailability of resveratrol, the level of resveratrol in the brain↓	Chen et al.
BBR	Male wistar rats and N2a cells	STZ-treated groups include DM, BBr, and Met were fed with high sugar and fat diet until the time for sacrifice.	N2a cell samples and hippocampal tissue	Glucose utilization, PI3k/PGC epsilon pathway↑; Aβ, AD-related neuronal damage↓	Wu et al.
BBR	Chinese hamster ovary (CHO) cells	CHO-APP695 cells were treated with BBR, PIO or a combination of BBR + PIO. Microglial cells were pre-treated with different concentrations of BBR, ranging from 0.3 to 10 μM and incubated for 2 h at 37°C.	Cell samples	basal respiration, pro-inflammatory cytokines in activated microglial cells↓	Wong et al.
BBR	A STZ diabetes rat model feeding with a high-fat diet	Metformin group with intragastrically administrated 0.18 g/kg of metformin hydrochloride and berberine group with intragastrically administrated 150 mg/kg of berberine once a day for 4 weeks.	Serum samples and hippocampus tissues	Cognitive function↑; IR, tau, neuronal apoptosis↓	Zhang et al.
BBR	Male Wistar rats	187.75 mg/kg/d of BBR for BBR group and 184 mg/kg/d of metformin for Met group	Brain tissues	IR, PI3K/Akt/mTOR, MAPK signaling pathways↓	Chen et al.
BBR	Male Sprague–Dawley rats	Alzheimer diabetic rats treated with BBR in a dose of 150 mg/kg and with metformin in dose of 540 mg/kg	Hippocampus tissues and blood samples	Endoplasmic reticulum (ER) stress, memory deficits↓	Xuan et al.
Extracts of P. harmala	Adult male Wistar rats	Normal rats in group III received daily i.p saline for 2 weeks followed by daily doses of P. harmala *per se* (187.5 mg/kg; p.o) for 4 weeks, lCl_3_-exposed rats in group IV were treated with the oral doses of P. harmala 30 min after each neurotoxin injection for a period of 4 weeks, starting from the 15th day of AlCl_3_ injection	Brain tissues	Aβ, p-tau, GSK3β↓	Saleh et al.
Hup A	HFD obese male C57 BL/6 mice and genetic ob/ob mice	Intragastrically administered 0.1 mg or 0.3 mg Hup A per kilogram of body weight per day or vehicle for three mouths.	Brain tissues	insulin, p-Akt↑in high fat-induced mice, but not seen in ob/ob mice	Wang et al.
Geniposide	Adult male C57BL/6J mice	Treatment groups with low (20 mg/kg, 4 mg/mL in saline) and high (100 mg/kg, 25 mg/mL in saline) by oral administration at the beginning of corticosterone treatment. CREB inhibitor 666–15 (20 mg/kg, 4 mg/mL in saline) was treated to 100 mg/kg geniposide treatment group together by I.P. injection.	Blood samples and hippocampus tissues	CREB↑; cognitive dysfunctions↓	Sun et al.
Geniposide	APPswe/PS1dE9 double transgenic AD mice (C57BL/6 background) and primary cortical neurons	Mice: Treated with geniposide (intragastric administration, i.g.) for consecutive 4 weeks at the dose of 5, 10, and 20 mg/kg, respectively. Liraglutide (100lg/kg, i.p.) was used as a positive control.	Blood samples, brain samples, and cell samples	p-Akt↑; P-tau, the phosphorylation of GSK-3β↓	Zhang et al.
		Cultured cells were incubated with 10 lM geniposide in the presence or absence of insulin (10 lM) for indicated times.			
Geniposide	APPswe/PS1dE9 double transgenic AD mice (C57BL/6 background) and Primary cortical neurons	Mice: Treated with geniposide (intragastric administration, i.g.) for consecutive 4 weeks at the dose of 5, 10, and 20 mg/kg, respectively. Liraglutide (100lg/kg, i.p.) was used as a positive control.	Brain samples and cell samples	BACE1, IDE↑	Zhang et al.
		Cultured cells were incubated with 10 lM geniposide in the presence or absence of insulin (10 lM) for indicated times.			
Sarsasapogenin	Male Sprague Dawley rats and SH-SY5Y cells	Rats: Treated with the low and high doses of Sar (20 and 60 mg/kg), and positive control insulin (5 U/kg). SH-SY5Y cells: low, middle, and high concentrations of Sar group (HG + Sar, 70 mmol/L glucose +0.1, 1, 10 μmol/L Sar, respectively), and positive control insulin group (HG + In sulin, 70 mmol/L glucose +50 mU/l insulin).	Brain and cell samples	BACE1, Akt/GSK-3β cascade↑; Aβ, p-tau↓	Zhang et al.
Asiaticoside	Adult male Sprague-Dawley rats and SH-SY5Y cells	Administered orally with asiaticoside (20 or 40 mg/kg), rosiglitazone (4 mg/kg), or vehicle (10 mL/kg distilled water instead) once daily (9:00 a.m.) for the consecutive 30 days. Pretreatment cells with asiaticoside (0.1, 1 μmol/L) and rosiglitazone (10 μmol/L) for 48 h	Blood samples, brain tissues, and cell samples	Synaptic function↑; cognitive deficits, oxidative stress, PI3K/Akt/NF-κB pathway↓	Yin et al.
Ginsenoside Rb1	C57BL/6N male mice	The ginsenoside Rb1 group (STZ+30 mg/kg ginsenoside Rb1).	Blood samples and brain tissues	Memory and cognitive ability, insulin sensitivity↑; Glucose intolerance↓	Yang et al.
Ginsenoside Rg2	5XFAD mice and HeLa cell lines	Treated with i.p. Injection of 20 mg/kg Rg2 (or DMSO vehicle) once/day, 5 days/week, for 16 weeks. Age-matched wild-type (WT) male mice were used as a negative control. HeLa cells were treated with using 100 ng/mL tetracycline, or 0.1 mM Rg2 with control or ATG7 siRNA for 48 h.	Blood samples, brain tissues, and cell samples	Cognitive behaviors, autophagy↑	Fan et al.
Quercetin	The db/db mice and age-matched wild-type C57BL/6J-db/m mice	db/db + low dosage of quercetin (QL, 35 mg/kg/d, n = 8) and db/db + high dosage of quercetin (QH, 70 mg/kg/d, n = 8).	Brain tissues	Cognition, brain-derived BDNF, Sirtuin type 1↑;insulin, fasting blood glucose↓	Hu et al.
Luteolin	Male C57BL/6J mice	The mice were randomized into four groups: Control diet (CD); control diet + luteolin (CDL, luteolin 10 mg/kg); high-fat diet (HFD); high-fat diet + luteolin (HFDL, luteolin 10 mg/kg)	Blood samples and brain tissues	BDNF, synapsin I, postsynaptic density protein 95↑; cognitive impairment, neuroinflammation, oxidative stress, neuronal insulin resistance↓	Liu et al.
Piperine	STZ induced rats	control (Vehicle only), diabetic control (STZ only), piperine treated (20 mg/kg day, i.p), and sitagliptin (Positive control) treated.	serum samples, pancreatic and brain tissue samples	Cognition↑; the expression of AD-associated genes↓	Kumar et al.
Artemisinin	Wistar rats	Intranasal administration of a single dose of TSP-1-hEDSCs and intraperitoneal administration of artemisinin for 4 weeks.	Blood samples and brain tissues	Aβ, glucose, ROS, TNF-α↓	Poorgholam et al.
Mangifera indica Linn extract (MGF)	The leptin receptor KO mouse db/db	9–12 mice per group (Control = 12, Control-Mangifera indica Linn extract (MGF) = 11, db/db = 10 and db/db-MGF = 9)	Brain tissues and blood samples	Central inflammation, atrophy, body weight, glucose, and insulin levels ↓	Infante-Garcia et al.
Lychee seed extract (LSE)	Male Sprague Dawley rats	Treated with normal saline (NS) 1 mL/kg (negative control), donepezil 0.42 mg/kg (positive control), LSE 0.7, 1.4 and 2.8 g/kg by intragastric (IG) administration once a day (daily) at 8 a.m. for 28 consecutive days.	Blood samples and hippocampus tissues	Cognitive functions↑; neuronal injury, glucose, insulin, Aβ, AGEs, tau↓	Tang et al.
Yuzu extract	Male Sprague-Dawley rats	AD control, AD yuzu, and control (d 7).	Brain tissues and blood samples	Hippocampal insulin signaling↓	Yang et al.
Luteolin, orientin, and isoorientin	RAW 264.7 cells	Treated with 5, 15, and 30 μM of luteolin, orientin, and isoorientin.	Cell samples	Isoorientin: DPPH, NO, and ONOO ↓	Choi et al.
				Luteolin: ROS, AChE, BChE, BACE1, PTP1B↓	
Vitexin, isovitexin, and apigenin	RAW 264.7 cells	Treated with various concentrations of vitexin, isovitexin, and apigenin (final concentration; 5, 15, and 30 lM).	Cell samples	Isovitexin: RLAR, HRAR, AGE, AChE, and BChE↓	Choi et al.
				Vitexin: PTP1B↓	
				Apigenin: NO production, iNOS and COX-2↓	

Symbol explanation: “↑” means upregulating, increasing, improving and so on. “↓” means downregulating, decreasing, impairing and so on.

**TABLE 3:** Overview of clinical trials of diabetic drugs and herbal medicines in treatment of AD.

Objective of study	Types of antidiabetic drugs	Types of clinical trials	Outcome characteristics	References
T2DM, dementia	Metformin	A population-based investigation	Metformin improved cognitive performance and decreased the risk of dementia	Cheng et al.
MCI due to AD	Metformin	A paired study	Patients with metformin had higher levels of Aβ and lower levels of tau and p-Tau in CSF. Metformin might reduce hippocampus and cortical atrophy to cognitive improvement.	Pomilio et al.
Abnormal glucose metabolism and NDVCI	Metformin	A randomized controlled trial	The performance function was increased. The levels of fasting insulin and IR index is significantly decreased in the metformin-donepezil group than that in the acarbose-donepezil group.	Lin et al.
AD, T2DM	Metformin	A population-based nested case-control study	Metformin was significantly associated with an increased risk of AD.	Ha et al.
AD, MCI, and DM	Metformin	A prospective research	Worse cognitive performance was associated with metformin use.	Moore et al.
AD	Exenatide	A double-blind randomized placebo-controlled Phase II clinical trial	Exenatide treatment produced no differences or trends compared to placebo for clinical and cognitive measures, MRI cortical thickness and volume, or biomarkers in CSF, plasma, and plasma neuronal EV except for a reduction of Aβ42 in EVs.	Mullins et al.
AD	Liraglutide	A randomized study	Liraglutide highly significantly raised the blood-brain glucose transfer capacity (T (max)) estimates of cerebral cortex compared to placebo.	Gejl et al.
AD	Liraglutide	A randomized, placebo-controlled double-blinded clinical trial	Liraglutide prevented the decline of glucose metabolism. There was no firm conclusions from the Aβ load or cognition measures.	Gejl et al.
Subjective cognitive complaints	Liraglutide	A placebo-controlled study	Significant improvement in intrinsic connectivity within the DMN was observed in liraglutide group relative to placebo by fMRI.	Watson et al.
Cognitive impairment in T2DM	Dulaglutide	A randomized, double-blind placebo-controlled trial	The hazard of substantive cognitive impairment was reduced by 14% in those assigned dulaglutide.	Cukierman-Yaffe et al.
MCI, T2DM	DPP4is	A population-based cohort study	DPP4 activity was negatively associated with BDNF. The risk for MCI increased with higher levels of DPP4 activity.	Zheng et al.
DM and AD-related cognitive impairment	DPP4is	A retrospective investigation	The DPP4is group had lower global amyloid burden, lower regional amyloid burden in temporo-parietal areas, and a slower longitudinal decrease in MMSE score and memory recall subscore.	Jeong et al.
Memory-impaired adults with AD or amnestic MCI	Insulin Therapy	A randomized controlled trial	Intranasal insulin improved verbal memory and acutely increased plasma Aβ42 levels.	Reger et al.
T2DM, AD	Curcumin	A 12-week randomized controlled trial	Participants with prediabetes who consumed 180 mg of curcumin daily had significantly reduced GSK3β and IAPP in serum samples after 12 weeks compared with placebo groups, reducing insulin resistance and the risk of developing AD and DM.	Thota et al.
/	Curcumin	A randomized, double-blind, placebo-controlled trial	Healthy older people with solid lipid curcumin have significantly better memory performance and mood.	Cox et al.
AD	Curcumin	A randomized, placebo-controlled, double-blind, pilot clinical trial	Curcumin can help patients with AD decrease the accumulation of Aβ in the brain, although Aβ levels in serum were no significant difference.	Baum et al.
/	Resveratrol	An interventional study	Supplementary resveratrol could improve memory function, accompanied by increased hippocampal functional connectivity and glucose metabolism.	Witte et al.
AD	Resveratrol	A randomized, double-blind, placebo-controlled trial	The treatment of resveratrol had no benefits in biomarkers of AD and glucose metabolism. And brain volume loss was increased in the resveratrol group.	Turner et al.
T2DM with normal cognition or AD	Metformin, DPP4is	A retrospective investigation	In normal cognition (NC) (n = 1192), metformin use was associated with better memory performance over time, whereas in AD (n = 807), DPP4is use was associated with a slower rate of memory decline. Interaction effects suggested greater benefit associated with DPP4is use among APOE epsilon4 carriers.	Wu et al.
T2DM and AD or mixed-pathology dementia	Metformin, insulin, sulfonylurea, thiazolidinediones (TZD), and DPP4is	A prospective open-cohort study	Compared to non-users, prevalent users of metformin and DPP4is experienced a slower cognitive decline with time. Secondly, compared to DPP4is, the use of insulin and sulfonylureas was associated with larger point-wise decrements in MMSE with annual intervals.	Secnik et al.
DM with or without AD	Sitagliptin, metformin, and insulin	A prospective and observational study	Besides its effects similar to those of insulin and metformin in glycemic control and in reducing need for insulin, 6-month sitagliptin therapy may also associated with improvement of cognitive function in elderly DM patients with and without AD.	Isik et al.
Dementia, T2DM	DPP4is, metformin, TZD, sulfonylurea, and insulin	A meta-analysis	Patients with T2DM under treatment with DPP4is presented with the lowest risk of dementia, followed by those treated with metformin and TZD, while treatment with insulin was associated with the highest risk.	Zhou et al.
Dementia	SGLT2is, DPP4is	A retrospective study	The use of SGLT2 is associated with lower risks of dementia compared with DPP4is use.	Mui et al.
Dementia	SGLT2is, DPP4is	A retrospective study	SGLT2is showed an association with lower dementia risk compared with DPP4is. Dapagliflozin exhibited the lowest risk, followed by empagliflozin, whereas canagliflozin showed no association.	Wu et al.

## 5 Discussion

The incidence and prevalence of Alzheimer’s disease, the most common form of dementia, have been rising rapidly over the past few decades. Although the pathology of AD is not fully understood, some studies focused on central metabolism including insulin signaling and mitochondria dysfunction have demonstrated the relationship between aberrant glucose metabolism and AD. Hyperglycemia is a major risk factor in the development of AD, and the central nervous system can also respond to glucose metabolism except for peripheral systems. Insulin can bind to insulin substrate through the BBB, thus mediating insulin signaling pathways to regulate energy metabolism and protect neurons. Impaired insulin signaling pathways, including PI3K/Akt/GSK3β and MAPK pathways, could cause damage to the brain in the pathogenesis of AD. Mitochondrial dysfunction and overexpression of TXNIP could also be causative links between AD and DM. These pathological processes do not exist independently, along with Aβ and phosphorylated tau. They can influence and interfere with each other to form a vicious circle.

Anti-diabetic medicines can also affect protein aggregation metabolism and neuroinflammation in the brain, which can dramatically enhance memory and cognition. Metformin has been shown to ameliorate AD, but results from multiple studies were inconsistent in rodent models. Some indicated that metformin is beneficial for cognitive decline and pathology of AD, but others argued that metformin might contribute to them because of vitamin B12 deficiency. The effect of Exendin-4 on AD is prominent in animal experiments, but more clinical trials are still lacking. Liraglutide as a promising drug for current treatment has confirmed its effect on AD in animal models and restored the decline of glucose metabolism in the brain in clinical trials. Semaglutide was proven to be effective in AD treatment with animal models and yet there is a lack of clinical trials. DPP4is such as saxagliptin, vildagliptin, linagliptin, and sitagliptin were effective in animal models. And only sitagliptin can enhance cognitive performance in current clinical trials. Compared to DPP4is, SGLT2is were considerably more protective in T2DM patients against new-onset dementia according to a retrospective study. In the existing studies of SGLT2is, empagliflozin, and dapagliflozin have neuroprotective effects on AD in animal models, and animals with dapagliflozin had the lowest chance of developing dementia in a retrospective study. The effects of DPP4is and SGLT2is still need more clinical trials to clarify. Insulin therapy is a promising therapy but some studies indicated that it may increase the risk of AD. To fully explore their potentials for treating AD, more research on the impact of antidiabetics on dementia risk is required, particularly in the clinical environment.

Moreover, the evidence in the text showed that herbal medicine also has effects on AD and diabetes. Plants and their extracts can improve cognition, and ameliorate the pathological changes associated with AD. Polyphenols like curcumin and resveratrol, BBR, glycosides such as geniposide and sarsasapogenin, and quercetin can correct metabolic abnormalities through PI3K/Akt/GSK3β and MAPK signaling pathways in AD models. Active metabolites of plants are the most promising component to apply for medicine formation, but their low bioavailability remains to be solved. Nanotechnology may offer broader prospects for the adoption of bioactive ingredients. More clinical trials are needed to verify the effectiveness of herbal medicines for AD based on animal studies.

## Glossary

3xTg-AD: triple transgenic mice model of AD
ACEI: angiotensin-converting enzyme inhibitors
AChE: acetylcholinesterases
AD: Alzheimer’s Disease
**AGEs**: advanced glycation end products
AMPK: AMP-activated protein kinase
APP: amyloid precursor protein
Aβ: β-amyloid
AβO: Amyloid beta oligomers
BACE1: beta-site APP cleaving enzyme
BBB: Blood-Brain Barrier
BBR: Berberine
BDNF: Brain-Derived Neurotrophic Factor
BuChE: butyrylcholinesterase
cGMP: cyclic guanosine monophosphate
CMA: Chaperone-mediated autophagy
CNS: central nervous system
CSF: cerebrospinal fluid
CurNP: curcumin nanoparticle
DA5-CH: GLP-1/GIP Dual-Agonist-5
DBR: BDNF ratio
DM: Diabetes Mellitus
**DMN**: default mode network
DPP4: dipeptidyl peptidase IV
DPP4is: dipeptidyl peptidase IV inhibitors
ER: endoplasmic reticulum
ERK: extracellular signal-regulated kinase
EV: extracellular vesicles
fMRI: functional magnetic resonance imaging
GFAP: glial fibrillary acidic protein
GLP-1: Glucagon-like peptide-1
**GLUT-1**: glucose transporter-1
GIP: Glucose-dependent insulinotropic polypeptide
GRU102: transgenic *Caenorhabditis elegans* with low levels of constitutive pan-neuronal expression of human Aβ1-42
GSK3β: glycogen synthase kinase 3β
Hup A: Huperzine A
IAPP: islet amyloid polypeptide
ICV-STZ: intracerebroventricular-streptozotocin
IDE: insulin-degrading enzyme
IMT: intima-media thickness
IRS-1: insulin receptors
IRS-1pSer: serine phosphorylation of IRS-1
JNK: c-Jun N-terminal kinase
L-LTP: late-phase long-term potentiation
LTP: long-term potentiation
MAPK: Mitogen-Activated Protein Kinase
MCI: moderate cognitive impairment
MMSE: Mini-Mental State Examination
mTOR: mammalian target of rapamycin
NDVCI: non-dementia vascular cognitive impairment
NFTs: intracellular neurofibrillary tangles
NF-κB: nuclear factor-kappa B
**NLRP3**: Nod-like receptor protein 3
PET: micro-positron emission tomography
PI3K: Phosphatidylinositol 3-kinase
PKA: protein kinase A
**PP**: antidiabetic polypill
PS1-KI: presenilin-1 knock-in
P-tau: phosphorylated tau
P. harmala: Peganum harmala
Ras: receptor agonists
RAGE: receptor for advanced glycation end products
ROS: reactive oxygen species
SGLT2is: sodium-glucose cotransporter 2 inhibitors
TA: triple GLP-1/GIP/glucagon receptor agonist
TCA: ricarboxylic acid cycle
Tg: transgenic
Tg6799: Transgenic mice
TR: target replacement
TrkB: tropomyosin-related kinase B receptor
TRX: thioredoxin
TXNIP: Thioredoxin-interacting protein
